# Supplementary material for: Early Lifestyle Interventions in People with Impaired Glucose Tolerance in Northern Colombia: The DEMOJUAN Project
Source: Int J Environ Res Public Health. 2019 Apr 18;16(8):1403. doi: 10.3390/ijerph16081403 (PMC6518277; doi:10.3390/ijerph16081403)
Supplement: Supplementary file 1 [file ijerph-16-01403-s001.zip › Appendix B.docx]

**Appendix B. Description of the topics of the interventions.**

The group seminars were developed with emphasis on nutrition and physical activity. Each workshop has its objectives and developed chronogram of the class activities including Power Point and class material. The development of the physical activity sessions during the class depends on each group. Overall, the study participants received educational materials and followed a predetermined order to monitor progress program.

The intervention was based on two approaches: (i) several individual appointments with a nutritionist and a physical activity expert and (ii) monthly group seminars consisting of the topics described below. The group sessions aimed at increasing knowledge and improve compliance by working together for a common goal.

**Description of the nutritional intervention**

A. Individual appointments with a nutritionist

During the first visit all personal data, family and personal history of diseases, as well as eating habits were evaluated using as basis the frequency of food consumption and the 24-hour recall questionnaires. With help of these questionnaires, calorie, grams of cholesterol, proteins, fat and fiber intake were assessed. In addition, the anthropometric measurements were performed. In the end, taking into account socio-economic status and the individual person data, a meal plan was set up including goals of the nutritional intervention. The recommendations were given based on the official nutritional recommendations of the region.

B. Group seminars

In the group seminars following topics were addressed:

- Healthy eating and energy needs
- Food and food groups
- Motivation
- Types of carbohydrates, fats and proteins
- Importance of fruit and vegetables
- Nutrition tips
- Tools for a healthy nutrition

At the beginning of each seminar the key messages of the previous seminars were repeated. The seminars consisted of participatory activities where the participants have to solve problems together and keep themselves motivated working as group in order to achieve better changes than the other groups (group competition).

**Group B: Topics of the nutritional intervention**

**Months 1-12**

| **Month 1** | **Month 2** | | | **Month 3** | **Month 4** | | | **Month 5** | | **Month 6** | |
| --- | --- | --- | --- | --- | --- | --- | --- | --- | --- | --- | --- |
| Project information | Food Components | | | Types of Carbohydrates, fats and proteins | Metabolism, vegetables, fruits | | | Healthy nutrition, energy balance | | Nutrition consulting | |
| Health and nutrition | Food Components | | | Cooking workshop | Cooking workshop | | | Cooking workshop | | Cooking workshop | |
| Visit with nutritionist | Cooking workshop | | |  | Visit with nutritionist | | |  | |  | |
|  |  | | |  |  | | |  |  |  |  |
| **Month 7** | **Month 8** | | | **Month 9** | **Month 10** | | | **Month 11** | | **Month 12** | |
|  | Safety of physical activity. | | | The benefits of physical activity. Warm up program for physical activity | Program “Dance yourself healthy”.  Summary of previous sessions. | | | Ecological walk. Summary of previous sessions. | | Physical activity in the swimming pool.  Summary of previous sessions. | |
| Definition of physical activity | Guided physical activity. | | | Guided physical activity. | Guided physical activity. | | | Guided physical activity. | | Guided physical activity. | |
| Physical activity assessment | Physical activity assessment | | |  | Physical activity assessment | | |  | |  | |
|  |  | | |  | Visit with nutritionist | | |  | |  | |
| **Months 13-24** | |  |  | | | |  | |  | |  |
| **Month 14** | **Month 16** | | | **Month 18** | | **Month 20** | | **Month 22** | | **Month 24** | |
| Improving nutritional habits  Cooking workshop | Improving nutritional habits  Cooking workshop | | | Improving nutritional habits  Cooking workshop | | Improving nutritional habits  Cooking workshop | | Improving nutritional habits  Cooking workshop | | Improving nutritional habits  Cooking workshop | |
| Guided physical activity. | Guided physical activity. | | | Guided physical activity. | | Guided physical activity. | | Guided physical activity. | | Guided physical activity. | |
|  | Physical activity assessment | | |  | | Physical activity assessment | |  | |  | |
|  | Visit with nutritionist | | |  | | Visit with nutritionist | |  | |  | |

###

### Group C: Physical Activity intervention

**Description of the physical activity intervention**

A. Individual appointment with a physical activity expert

During the first visit all personal data, family and personal history of diseases, as well as physical condition of the person were assessed, including physical activity behavior, past injuries due to physical activity such as lower limb osteoarthritis, sprains, rheumatoid arthritis, lumbago, dizziness, headache, migraine, cardio-respiratory problems. Additionally, the maximum heart rate was measured to determine VO2max and to establish a baseline for the physical activity interventions. Furthermore, in all intervention participants a 6-minut walk test was performed to measure aerobic cardio-respiratory capacity. Tests for strength and flexibility were applied as well. Anthropometric measures were taken as it is in the nutritional intervention group and are created indicators to confirm that decrease the metabolic risks, and achieve. A concentration test, to measure the degree of concentration and level of retention of knowledge was conducted and breathing techniques taught in order to correct inhalation and exhalation. The participant received information regarding warming-up and stretching before and after physical activity sessions.

B. Group seminars

The group seminars consisted of topics such as the benefits of physical activity, followed by group recreational physical activities emphasizing in generating commitments for being physically active. At the beginning of each seminar the key messages of the previous seminars were repeated. The seminar consisted of participatory activities where the participants had to solve problems together and keep themselves motivated working as a group in order to achieve better changes than the other groups (group competition).

**Months 1-12**

|  |  | |  | | |  | |  | |  |  |
| --- | --- | --- | --- | --- | --- | --- | --- | --- | --- | --- | --- |
| **Month 1** | **Month 2** | | **Month 3** | | | **Month 4** | | **Month 5** | | **Month 6** |  |
|  | Safety of physical activity. | | The benefits of physical activity. Warm up program for physical activity | | | Program “Dance yourself healthy”.  Summary of previous sessions. | | Ecological walk. Summary of previous sessions. | | Physical activity in the swimming pool.  Summary of previous sessions. |  |
| Definition of physical activity | Guided physical activity. | | Guided physical activity. | | | Guided physical activity. | | Guided physical activity. | | Guided physical activity. |  |
| Physical activity assessment | Physical activity assessment | |  | | | Physical activity assessment | |  | |  |  |
|  |  | |  | | | Visit with nutritionist | |  | |  |  |
|  |  | |  | | |  | |  | |  |  |
| **Month 7** | **Month 8** | | **Month 9** | | | **Month 10** | | **Month 11** | | **Month 12** |  |
| Project information | Food Components | | Types of Carbohydrates, fats and proteins | | | Metabolism, vegetables, fruits | | Healthy nutrition, energy balance | | Nutrition consulting |  |
| Health and nutrition | Food Components | | Cooking workshop | | | Cooking workshop | | Cooking workshop | | Cooking workshop |  |
| Visit with nutritionist | Cooking workshop | |  | | | Visit with nutritionist | |  | |  |  |
| **Months 13-24** | |  | |  |  | |  | |  | | |
|  | |  | |  |  | |  | |  | | |
| **Month 14** | | **Month 16** | | **Month 18** | **Month 20** | | **Month 22** | | **Month 24** | | |
|  | |  | |  |  | |  | |  | | |
| Improving nutritional habits  Cooking workshop | | Improving nutritional habits  Cooking workshop | | Improving nutritional habits  Cooking workshop | Improving nutritional habits  Cooking workshop | | Improving nutritional habits  Cooking workshop | | Improving nutritional habits  Cooking workshop | | |
| Guided physical activity. | | Guided physical activity. | | Guided physical activity. | Guided physical activity. | | Guided physical activity. | | Guided physical activity. | | |
|  | | Physical activity assessment | |  | Physical activity assessment | |  | |  | | |
|  | |  | |  | Visit with nutritionist | |  | |  | | |
