# Supplementary material for: Early Lifestyle Interventions in People with Impaired Glucose Tolerance in Northern Colombia: The DEMOJUAN Project
Source: Int J Environ Res Public Health. 2019 Apr 18;16(8):1403. doi: 10.3390/ijerph16081403 (PMC6518277; doi:10.3390/ijerph16081403)
Supplement: Supplementary file 1 [file ijerph-16-01403-s001.zip › Appendix A.docx]

| **Codigo de identification Barranquilla** | | | |
| --- | --- | --- | --- |
| **Area** | **Centre** | **Investigator Nº** | Cuestionario Nº |

**TEST DE FINDRISC**

Elija la opción correcta y sume los puntos obtenidos:

**1. Edad: _______Años**

O Menos de 45 años (0 p.)

O 45-54 años (2 p.)

O 55-64 años (3 p.)

O Más de 64 años (4 p.)

**2. Índice de masa corporal**

(Calcule su índice, según el apartado al final del test):

**Peso: ________Talla: ________IMC:__________**

O Menor de 25 kg/m^2^ (0 p.)

O Entre 25-30 kg/m^2^ (1 p.)

O Mayor de 30 kg/m^2^ (3 p.)

**3. Perímetro de cintura** medido por debajo de las costillas (normalmente a nivel del ombligo): **___________cm**

**Hombres Mujeres**

O Menos de 94 cm. O Menos de 80 cm. (0 p.)

O Entre 94-102 cm. O Entre 80-88 cm. (3 p.)

O Más de 102 cm. O Más de 88 cm. (4 p.)

4. ¿Realiza habitualmente al menos 30 minutos de actividad física, en el trabajo y/o en el tiempo libre?:

O Sí (0 p.)

O No (2 p.)

**5. ¿Con qué frecuencia come verduras o frutas?:**

O Todos los días (0 p.)

O No todos los días (1 p.)

**6. ¿Toma medicación para la hipertensión regularmente?:**

O No (0 p.)

O Sí (2 p.)

7. ¿Le han encontrado alguna vez valores de glucosa altos (Ej. en un control médico, durante una enfermedad, durante el embarazo)?:

O No (0 p.)

O Sí (5 p.)

8. ¿Se le ha diagnosticado diabetes (tipo 1 o tipo 2) a alguno de sus familiares allegados u otros parientes?

O No (0 p.)

O Sí: abuelos, tía, tío, primo hermano (no padres, hermanos o hijos) (3 p.)

O Sí: padres, hermanos o hijos (5 p.)

# Escala de Riesgo Total

**PARTICIPANTES CON PUNTUACION FINAL MAYOR O IGUAL A TRECE P (13) CITAR PARA REALIZARLE PARACLINICOS (PTOG, PERFIL LIPIDICO y CUESTIONARIO ADDICIONAL)**

*ÍNDICE DE MASA CORPORAL*

*El índice de masa corporal se usa para evaluar si una persona se encuentra en su peso normal o no. Se calcula dividiendo su peso (en kg) por el cuadrado de su estatura (en m). Por ejemplo, si su estatura es de 1.65 m y su peso de 70 kg, su índice de masa corporal será :*

***70/(1.65 x 1.65) = 25.7.***

*Si su índice de masa corporal se encuentra entre* ***25-30****, se beneficiaría si perdiera peso; o al menos tendría que vigilar que su peso no aumentara más. Si su* ***índice de masa corporal*** *es* ***mayor de 30,*** *los efectos adversos de la obesidad en su salud pronto se manifestarán, y sería esencial empezar a perder peso.*

**Cuestionario para personas con FINDRISC mayor o igual a 13 puntos**

# A-DATOS TÉCNICOS

**Puntaje del cuestionario FINDRISC:_________ Encuestador:________________**

| **Se ha leído y obtenido el consentimiento al entrevistado** | **1 Si**  **2 No** | **Estado civil** | **1 Casado**  **2 Unión Libre**  **3 Solero**  **4 Separado o divorciado**  **5 Viudo** |
| --- | --- | --- | --- |
| **Nivel educativo** | **1 Primaria**  **2 Secundaria**  **3 Técnico**  **4 Universidad** | **Profesión:** |  |
| **Nombre de la ARS o EPS a la que pertenece** |  | | |

## Sección 1. Cobertura y acceso a la atención médica

**1 ¿Qué cobertura de salud tiene?**

1 SISBEN (no afiliado a ARS)

2 Asegurado por ARS

3 Asegurado por EPS (TRABAJO)

4 Otro

##

## Sección 2. Tabaco

**2.1 ¿Has fumado alguna vez?**

1 Sí

2 No (pase a Sección 3)

**2.2 ¿Fumas actualmente?**

1 No en la actualidad

2 Si, ocasionalmente

3 Si, frecuentemente

**2.3 ¿Recuerda cuanto tiempo hace que fuma?**

1 En años ____________

2 en meses _________

3 en semanas _______

**2.4 Si usted fuma frecuentemente en promedio, ¿cuántos cigarrillos, cigarros, pipa u otras formas de tabaco fumó por día?**

1 Número de cigarrillos: _____

2 Tabaco de enrollar: _______

3 Pipas: _______

**2.5 ¿Cuándo fue la última vez que fumó?**

1 Hace 1 día o 1 mes

2 de 1 a 6 meses

3 Entre 6 y 12 meses

4 Entre 1 y 5 años

5 Más de 5 años

##

## Sección 3. Hipertensión arterial

(Ahora le haré algunas preguntas sobre su presión arterial)

**3.1 ¿Alguna vez un médico, una enfermera o otro profesional de la salud le dijo que tenía la presión alta?**

1 Sí, sólo una vez

2 Sí, más de una vez

3 No (Pase a Sección 4)

4 No recuerda (pase a Sección 4)

**3.2 ¿En estos momentos está haciendo algún tratamiento**

**(medicamentos, dieta, ejercicio) indicado por un profesional de la salud para controlar su presión arterial?**

1 Sí

2 No (pase a Sección 4)

**3.3 ¿Qué tipo de tratamiento está haciendo?**

Medicamentos durante las ultimas dos semanas **si/no**

Dieta especial para disminuir el consumo de sal **si/no**

Para perder peso **si/no**

Para dejar de fumar **si/no**

Mas ejercicio **si/no**

Medicamento tradicional o a base de hierbas para su tensión alta

**si/no**

## Sección 4. Actividad física (Ahora hablaremos sobre la cantidad y el tipo de actividad física que Ud. hace cotidianamente)

**4.1 ¿Su trabajo es de gran exigencia física? La actividad en el trabajo se divide en 4 grupos. Trabajo significa actividades cotidianas, empleo o trabajo sin sueldo.**

1 En mi trabajo **estoy principalmente sentado** y no camino demasiado en horario laboral (por ejemplo relojero, operador de radio, costurero/a, oficinista)

2 **Camino bastante en mi trabajo,** pero no levanto ni llevo objetos pesados (por ejemplo encargado y ayudante de almacén, trabajador de pequeña industria, trabajo de oficina que requiere caminar).

3 He de **caminar mucho y cargo o subo escaleras** o pendientes diariamente (por ejemplo carpintero o pastor, taller industrial u otros t rabajos industriales pesados).

4 Mi labor conlleva un **trabajo manual duro** pues tengo que levantar o llevar objetos pesados, cavar, excavar o talar (por ejemplo trabajo forestal, trabajo duro de granja, trabajo duro de construcción o industria).

**4.2 ¿Cuanto ejercicio o actividad física realiza en su tiempo libre? Si**

**varia en las diferentes estaciones del año señalar la mejor**

**alternativa que describa su situación promedio.**

1 En mi tiempo libre leo, veo la televisión y hago algún trabajo doméstico que no requiere mucho esfuerzo

2 En mi tiempo libre paseo, voy en bicicleta o hago ejercicio al menos 4 horas por semana. Eso incluye caminar, pescar o cazar, pero excluye el desplazamiento al trabajo.

3 En mi tiempo libre hago ejercicio para mantener mi condición física, por ejemplo correr, footing, esquiar, nadar, gimnasia, fútbol, senderismo o similares al menos 3 horas por semana.

4 En mi tiempo libre practico habitualmente, varias veces por semana, deportes de competición como carreras, artes marciales, esquí, natación, fútbol u otros deportes pesados.

##

**4.3 ¿Como considera su condición física actual?**

1 Muy buena

2 Bastante buena

3 Suficiente

4 Bastante mala

5 Muy mala

**4.4 ¿Ha aumentado su actividad física durante el último año?**

1 No lo he hecho / No tengo intención de aumentarla en un futuro próximo

2 No lo he hecho pero la aumentaré en un futuro próximo

3 He probado de aumentarla

4 La he aumentado

5 Ya hago mucha actividad física

## Sección 5. Alimentación

**5.3 ¿Le agrega sal a los alimentos una vez que están cocidos o al sentarse a la mesa?**

1 No, nunca

2 Rara vez

3 Algunas veces

4 Frecuentemente

5 Siempre

**5.2 ¿Qué usa más frecuentemente para cocinar (aceite, manteca, grasa)?**

1 Aceite vegetal

2 Aceite de oliva

3 Aceite de palma

4 Grasa

5 Manteca de animal

6 Mantequilla

7 Otro

8 Cocina sin ningún tipo de aceite o grasa

**5.1.1 Come usted frutas a diario (todos los días de la semana**

1. Si
2. No

**5.1.2 Come usted verduras a diario (todos los días de la semana**

1. Si
2. No

**5.6 ¿Hace Ud. algo regularmente para controlar su ingesta de sal o de sodio?**

1 Si

2 No (pasa a sección 6)

3 No sabe

4 Rehúsa responder

**5.7 ¿Que es lo que Ud. Hace?**

*(por favor, deja participante responder, no leer las opciones)*

1 Evita/minimiza el consumo de alimentos procesados

2 Revisa las etiquetas de sal o sodio en la comida

3 No agrega sal en la mesa

4 Compra alternativas con baja cantidad de sal

5 Compra alternativas con baja cantidad de sodio

6 No agrega sal a las comidas cuando las cocina

7 Cuando cocina utiliza otras especies en lugar de sal

8 Evita comer afuera de casa

9 Otro (especifique)________

**5.4 ¿A la comida que se prepara en casa se le agrega sal al momento de cocinarla?**

1 No, nunca

2 Rara vez

3 Algunas veces

4 Frecuentemente

5 Siempre

**5.5 ¿Cuánta sal consume Ud.?**

1 Muchísima

2 Mucha

3 Solo la cantidad justa

4 Poco

5 Muy poco

6 No sabe

## Sección 6. Diabetes

**(Ahora hablaremos sobre el nivel de azúcar en la sangre)**

**6.1 ¿Alguna vez un doctor, una enfermera u otro profesional de la salud le dijo que tenía diabetes o azúcar alta en la sangre?**

1 Si (Si es varón pase a pregunta 6.3)

2 No (pase a pregunta 6.5)

3 No recuerda (pase a pregunta 6.5)

**6.2 ¿Eso ocurrió cuando estaba embarazada?** *SOLO PARA MUJERES*

1 Sí

2 No

3 No recuerda

**6.3 ¿En estos momentos está haciendo algún tratamiento (medicamentos, dieta, ejercicio) indicado por un profesional de la salud para mantener controlada su diabetes / azúcar alta en sangre?**

1 Sí

2 No (pase a pregunta 6.5)

**6.4 ¿Qué tipo de tratamiento está haciendo?**

1 Medicamentos (pase a pregunta 6.6)

2 Tratamiento sin medicamentos (dieta, ejercicios, peso) (pase a pregunta 6.6)

3 Ambos (pase a pregunta 6.6)

**6.5 ¿Alguna vez le han medido la glucemia / azúcar en**

**Sangre?**

1 Sí

2 No (pase a Sección 7)

3 No recuerda

**6.6 ¿Cuándo fue la última vez que le midieron glucemia /**

**Azúcar en sangre?**

1 Menos de 1 año 1

2 Entre 1 y 2 años 2

3 Más de 2 años 3

4 No recuerda

## Sección 7. Historia personal y familiar

**(Ahora hablaremos un poco sobre el tema de historia familiar)**

**7.1 ¿Usted ha sido diagnosticado con las siguientes enfermedades?**

7.1a Derrame cerebral si/no

7.1b Cáncer o tumor maligno si/no

7.1c Infarto cardiaco temprano si/no

7.1d Angina pectoris si/no

**7.2 ¿Alguno de los miembros de su familia (consanguíneos) ha sido diagnosticado con las siguientes enfermedades?**

7.2a Presion alta si/no

7.2b Derrame cerebral si/no

7.2c Cáncer o tumor maligno si/no

7.2d Colesterol alto si/no

7.2e Infarto cardiaco temprano si/no

7.2f Angina pectoris si/no

# B-DATOS CLINICOS

Fecha: __ __/__ __/20__ __

**B1. Se ha completado y comprobado el cuestionario anterior.**

1. Sí ( )

2. No ( )

**B2. Talla (centímetros)**

**B3. Peso (kilogramos)**

|  |  |  | **,** |
| --- | --- | --- | --- |

**B4. Circunferencia de la cintura (centímetros)**

|  |  |  | **,** |
| --- | --- | --- | --- |

**B5. Presión arterial (mmHg)**

1. Primera medida

Sistólica Diastólica

1. Segunda medida

Sistólica Diastólica

3. Frecuencia cardiaca: latidos x min

**B6. Prueba oral de tolerancia a la glucosa (PTOG)**

1. OGTT se ha tomado en el plazo de un año, o han referido a la persona la prueba. Marque abajo la fecha de PTOG y de los resultados (pregunta B7.1)

2. El participante no desea realizar PTOG

**B7. Fecha de OGTT (d/m/y, e.g. 101005) y valores (muestra de plasma/venosa)**

Día mes año

|  | **B7.2 Valor de la glucosa**  **0-h (mg x dl)** | **B7.3 Valor de la glucosa**  **2-h (mg x dl)** |
| --- | --- | --- |
| **Muestra venosa** |  |  |

**B8. Medida lípidos en sangre:**

1. La medida de lípidos en sangre se ha tomado en el plazo de un año, o han referido a la persona la prueba. Marque abajo la fecha de la prueba y los resultados.

2. El Paciente no desea realizarse prueba de lípidos.

**B8.1 Fecha de la medida de los lípidos en sangre (dd-mm-yyyy) y de los resultados.**

Día mes año  **B9.2 Ayuno** Si____ No____

|  | **Valor (mg x dl)** |
| --- | --- |
| **B8.3 Colesterol total** |  |
| **B8.4 HDL-Colesterol** |  |
| **B8.5 LDL-Colesterol** |  |
| **B8.6 Triglceridos** |  |
